# Mixed Graphite/Carbon Black Recycled PLA Conductive Additive Manufacturing Filament for the Electrochemical Detection of Oxalate

**DOI:** 10.1021/acs.analchem.3c03193

**Published:** 2023-09-28

**Authors:** Iana V. S. Arantes, Robert D. Crapnell, Elena Bernalte, Matthew J. Whittingham, Thiago R. L. C. Paixão, Craig E. Banks

**Affiliations:** † Faculty of Science and Engineering, Manchester Metropolitan University, Chester Street, Manchester M1 5GD, U.K.; ‡ Departmento de Química Fundamental, Instituto de Química, Universidade de São Paulo, São Paulo, SP 05508-000, Brazil

## Abstract

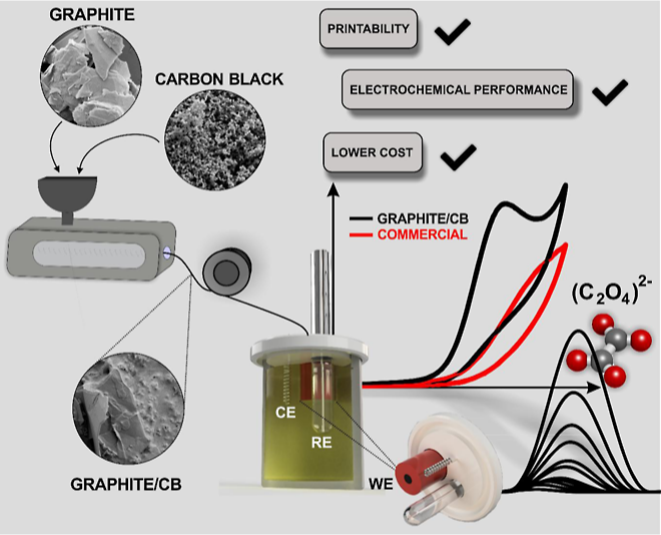

Mixing of graphite and carbon black (CB) alongside recycled poly(lactic acid) and castor oil to create an electrically conductive additive manufacturing filament without the use of solvents is reported herein. The additively manufactured electrodes (AMEs) were electrochemically benchmarked against a commercial conductive filament and a bespoke filament utilizing only CB. The graphite/CB produced a heterogeneous rate constant, *k*^0^, of 1.26 (±0.23) × 10^–3^ cm s^–1^ and resistance of only 155 ± 15 Ω, compared to 0.30 (±0.03) × 10^–3^ cm s^–1^ and 768 ± 96 Ω for the commercial AME. Including graphite within the filament reduced the cost of printing each AME from £0.09, with the CB-only filament, to £0.05. The additive manufacturing filament was successfully used to create an electroanalytical sensing platform for the detection of oxalate within a linear range of 10–500 μM, achieving a sensitivity of 0.0196 μA/μM, LOD of 5.7 μM and LOQ of 18.8 μM was obtained. Additionally, the cell was tested toward the detection of oxalate within a spiked synthetic urine sample, obtaining recoveries of 104%. This work highlights how, using mixed material composites, excellent electrochemical performance can be obtained at a reduced material cost, while also greatly improving the sustainability of the system.

## Introduction

Oxalate (C_2_O_4_^2–^) is a product of protein metabolism excreted by the kidneys and removed from humans via urine, therefore, the presence of oxalate within urine can be an indicator of kidney lesions such as renal failure and pancreatic insufficiency, among others.^1,2^ Additionally, an increased urinary oxalate concentration can lead to the development and formation of calcium oxalate kidney stones; the urinary oxalate concentration should not exceed 460 μM over a 24 h period.^3^ The detection of oxalate has successfully been reported using a wide range of classic benchtop methodologies, such as fluorescence,^4^ luminescence,^5^ liquid chromatography–mass spectrometry,^6^ and microchip electrophoresis.^7^ These methodologies typically require sample collection followed by transport to a laboratory, where the sample is analyzed using these benchmark tests by a skilled user. To improve patient care, real-time measurements of key analytes are sought after. Electroanalytical sensing platforms can realize this due to their ability to gather highly sensitive, selective, and fast measurements while being easily miniaturized and low cost.

Within electrochemical research additive manufacturing (AM), especially fused filament fabrication (FFF), is seeing a surge in popularity due to its many benefits,^8,9^ such as low cost of entry with good quality printers available for only a few hundred pounds and commercial conductive filament available for less than one hundred pounds for 500 g; rapid prototyping capabilities and in situ production; capability to explore various electrode geometries without high manufacturing costs;^10−12^ and low waste production due to its layer-by-layer additive approach to production, when compared to classical subtractive manufacturing methodologies. Within the electrochemical field, AM has been used to create bespoke devices, equipment,^9,12^ and electrodes. Initially, the designs of electrodes took the form of simple discs or lollipops,^13,14^ but have progressed to include full devices,^15^ with some designs embedding the electrodes within the cell to produce the product within a single print.^16^

Most additively manufactured electrodes (AMEs) are a single-use item due to difficulties replenishing the surface of the electrode and the ingress of solution within the polymer matrix.^17^ The use of single-shot electrodes is attractive in healthcare monitoring settings due to the necessity to avoid cross contamination between samples or patients. However, this leads to massive amounts of waste within the sector, with an estimated 1.6 million tons of plastic waste a day being consumed during the COVID-19 pandemic.^18^ The sustainability of practices has become increasingly important, with the United Nations outlining 17 sustainable goals for development globally.^19^ Goal 12 of this initiative is to “ensure sustainable consumption and production patterns”, which is challenging for areas that require single-use items. To help improve the sustainability of AM electrochemistry, Sigley et al.^20^ produced bespoke conductive FFF filament from recycled coffee machine plastic pod feedstock that later was successfully applied for the electrochemical detection of caffeine. Further improvements in the sustainability of conductive filament production were made through the introduction of castor oil as a biobased plasticizer.^21^ Additionally, it has recently been shown how used AM electroanalytical sensing platforms can be recycled into a new filament (both conductive and nonconductive) for the production of sensors.^22^

Within the work above, carbon black (CB, Super P), was added as a conductive filler with a loading of ∼25 wt %, increasing the amount found in the commercial conductive filament by ∼4.5 wt %.^23^ CB (Super P) is produced from the partial oxidation of petrochemical precursors and costs approximately £300 per 100 g for the specific grade used.^20^ On the other hand, graphite is naturally occurring and the most stable form of carbon under standard conditions, with a cost roughly 1% of the CB used above. Due to its good thermal stability, corrosion resistance, high specific strength, and electrical conductivity, graphite is commonly used to produce electrodes for electrochemical applications, such as within ink formulations to produce screen-printed electrodes.^24^ While a conductive graphite/poly(lactic acid) (PLA) filament has been reported in the literature, it had a resistance of 6 kΩ cm^–1^, showed poor performance when compared to nanocarbon and commercial options, and was produced using multiple steps and dichloromethane.^25^

In this work, we propose the development of conductive filament for FFF through a composite of CB and graphite in recycled PLA with the biobased plasticizer castor oil to increase the low-temperature flexibility of the filament. Utilizing a single mixing process, the polymer is subjected to minimal thermal steps for filament production and removes the use of harsh chemicals such as dichloromethane seen in previous reports.^25,26^ This work highlights how using mixed carbon material composites can create conductive filament with enhanced electrochemical properties and significantly lower the material cost while using recycled polymer and biobased plasticizers to improve the sustainability of the process and the use of additive manufacturing allows for bespoke cell designs to be produced for specific applications.

## Experimental Section

### Chemicals

Hexaamineruthenium(III) chloride (98%), castor oil, potassium ferricyanide (99%), potassium ferrocyanide (98.5–102%), sodium hydroxide (>98%), sodium oxalate (≥99.5%), potassium chloride (99.0–100.5%), sodium sulfate (≥99.0%), ammonium chloride (≥99.5%), potassium phosphate monobasic (≥99.0%), calcium chloride (≥96.0%), sodium chloride (≥99.0%), graphite powder (<20 μm), and phosphate-buffered saline (PBS) tablets were purchased from Merck (Gillingham, UK). CB (Super P, >99+%), urea (98+%), creatinine (98+%), and hydrochloric acid (37% ACS grade) were purchased from Fisher Scientific (Loughborough, UK). Recycled PLA was purchased from Gianeco (Turin, Italy). Commercial conductive PLA/CB filament (1.75 mm, ProtoPasta, Vancouver, Canada) was purchased from Farnell (Leeds, UK). Recycled nonconductive PLA filament was produced in-house as previously reported.^20^ All solutions were prepared with deionized water of resistivity not less than 18.2 MΩ cm from a Milli-Q Integral 3 system from Millipore UK (Watford, UK).

### Recycled Conductive Filament Production

Recycled PLA was dried in an oven at 60 °C for a minimum of 2.5 h before any mixing or filament production. The polymer composition was prepared using 65 wt % rPLA, 10 wt % castor oil, 15 wt % CB, and 10 wt % graphite powder. These are mixed (190 °C) with Banbury rotors (70 rpm for 5 min) using a Thermo Haake Poydrive dynameter fitted with a Thermo Haake Rheomix 600 (Thermo-Haake, Germany). This is allowed to cool to room temperature before being granulated to create a finer granule size using a (Rapid Granulator 1528). This is next processed through an EX6 extrusion line (Filabot, VA, United States) using a single screw with heat zones of 60, 190, 195, and 195 °C, respectively, which are extruded from a 1.75 mm die head. Then, the filament is ready to use for Additive Manufacturing (AM).

### Additive Manufacturing

Computer designs are produced using Fusion 360 (Autodesk, CA, United States). These files were sliced and converted to .GCODE files, which are printed by open-source software, PrusaSlicer (Prusa Research, Prague, Czech Republic). The AMEs were 3D-printed using FFF technology on a Prusa i3MK3S+ (Prusa Research, Prague, Czech Republic). All AMEs were printed using a 0.6 mm nozzle with a nozzle temperature of 215 °C, 100% rectilinear infill, 0.15 mm layer height, and print speed of 70 mm s^–1^. Details of the physiochemical characterization and electrochemical experiments are reported in the Supporting Information.

A synthetic urine sample was prepared according to Laube et al.,^27^ where 0.28 g CaCl_2_, 0.73 g NaCl, 0.35 g KH_2_PO_4_, 0.40 g KCl, 0.25 g NH_4_Cl, 0.56 g Na_2_SO_4_, 6.25 g urea, and 0.28 g creatinine were dissolved in 250 mL of ultrapure water. The pH was adjusted to 6.0 using a 1.0 M HCl solution. The sample was first spiked with 500 μmol L^–1^ sodium oxalate salt and then diluted (∼20-fold) in the supporting electrolyte for a standard addition study.

## Results and Discussion

### Production and Characterization of Recycled Filament

**Figure 1:**
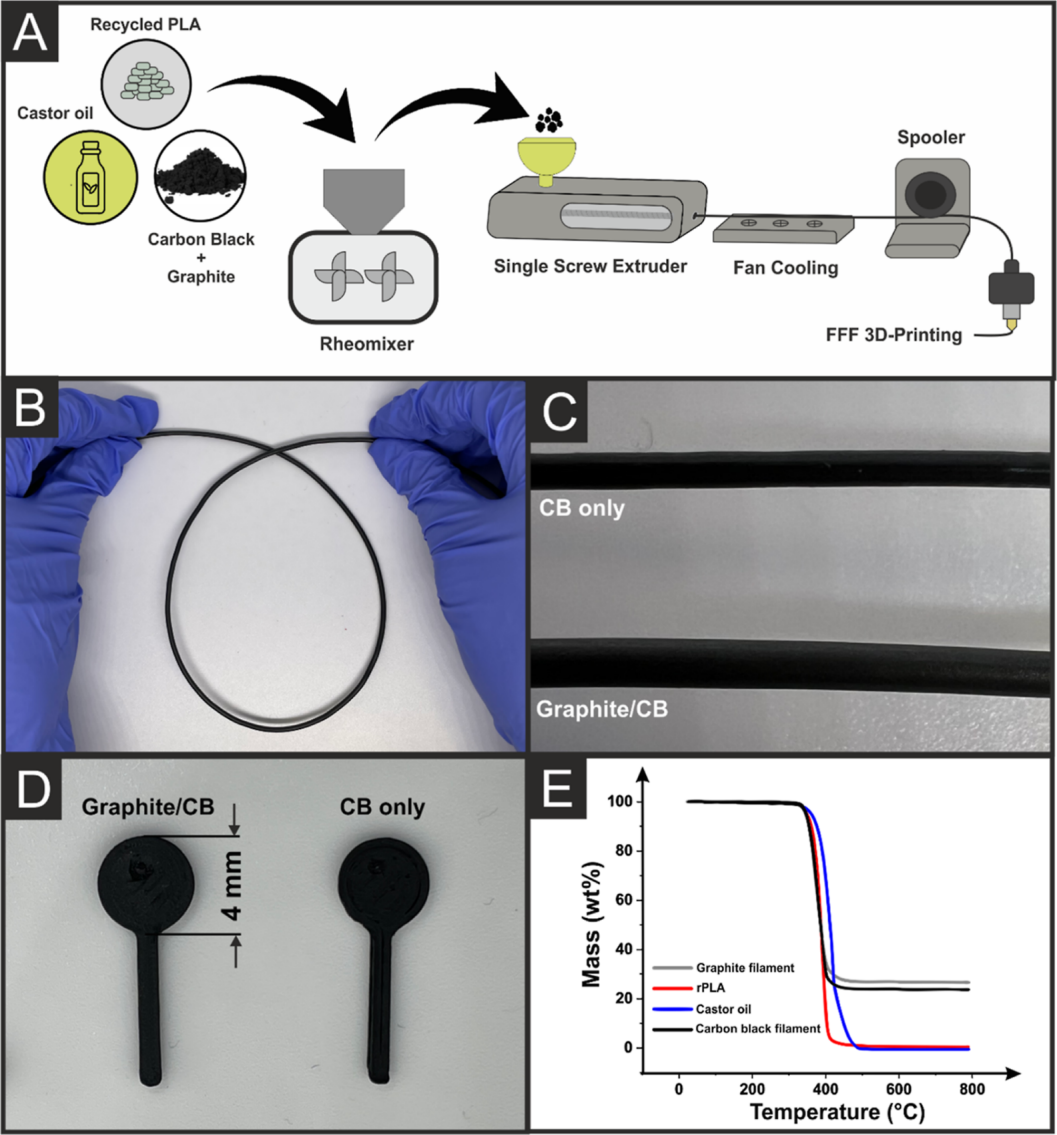
(A) Schematic representation of filament production. (B,C) Images of the bespoke graphite/CB filament showing (B) its flexibility and (C) comparison with the bespoke CB-only filament. (D) Images of the lollipop AMEs printed from the bespoke graphite/CB and the CB/only filament. (E) Thermal gravimetric analysis of graphite, recycled PLA, castor oil, and CB.

The production of additive manufacturing filament from recycled PLA (rPLA), castor oil, CB (Super P), and graphite was achieved in the same way as reported previously and is presented in Figure 1A.^21^ This methodology is a single thermal mixing step, which removes any requirement for additional solvents seen within other reported methods.^25,26^ The filament produced had excellent room-temperature flexibility, as shown in Figure 1B and a shiny, silver quality to the surface of the graphite/CB filament compared to the dark matt black finish of the original CB filament due to the presence of the graphite, Figure 1C. The graphite/CB filament exhibited excellent printability, as shown for the lollipop-shaped AMEs, Figure 1D, where no additional extrusion rate was required to get a high-quality print. In comparison, the CB-only filament required increased extrusion rates to achieve a nonporous print surface.^21^

The graphite/CB filament gave a resistance across 10 cm of the filament of 875 ± 38 Ω, which is statistically the same as the filament using only CB reported previously^21^ and was a significant improvement upon the commercial conductive filaments reported values of 2–3 kΩ. The significant improvement in the commercial system is primarily due to the quality of the conductive filler and increased carbon loading. Thermogravimetric analysis of the graphite/CB filament, Figure 1E, indicated a filler level of 24 ± 1 wt %. This was calculated through the stabilization of the thermogravimetric analysis (TGA) curve after the degradation of rPLA and castor oil. This value is more consistent when compared to data obtained previously for only CB filament, which is attributed to the higher density of the graphite powder, meaning there is less material lost when filling the mixing devices. The onset temperature of thermal degradation and conductive filler contents, where applicable, are presented in Table S1. It can be seen that the rPLA used throughout this study had an average onset of degradation temperature of 304 ± 4 °C, which showed good agreement with previous work.^20,28^ As previously reported, castor oil produced an onset temperature of 250 ± 3 °C.^21^ The bespoke graphite/CB filament produced an average onset of degradation temperature of 283 ± 4 °C, which indicated that the conductive carbon fillers provided a stabilizing effect by acting as a barrier for gas diffusion out of the polymer, slowing the rate of decomposition.^29^ The onset temperature seen for the graphite/CB filament is similar to that seen for the CB only filament published previously,^21^ indicating that replacing some CB with graphite is not detrimental to the thermal stability of the filament.

**Figure 2:**
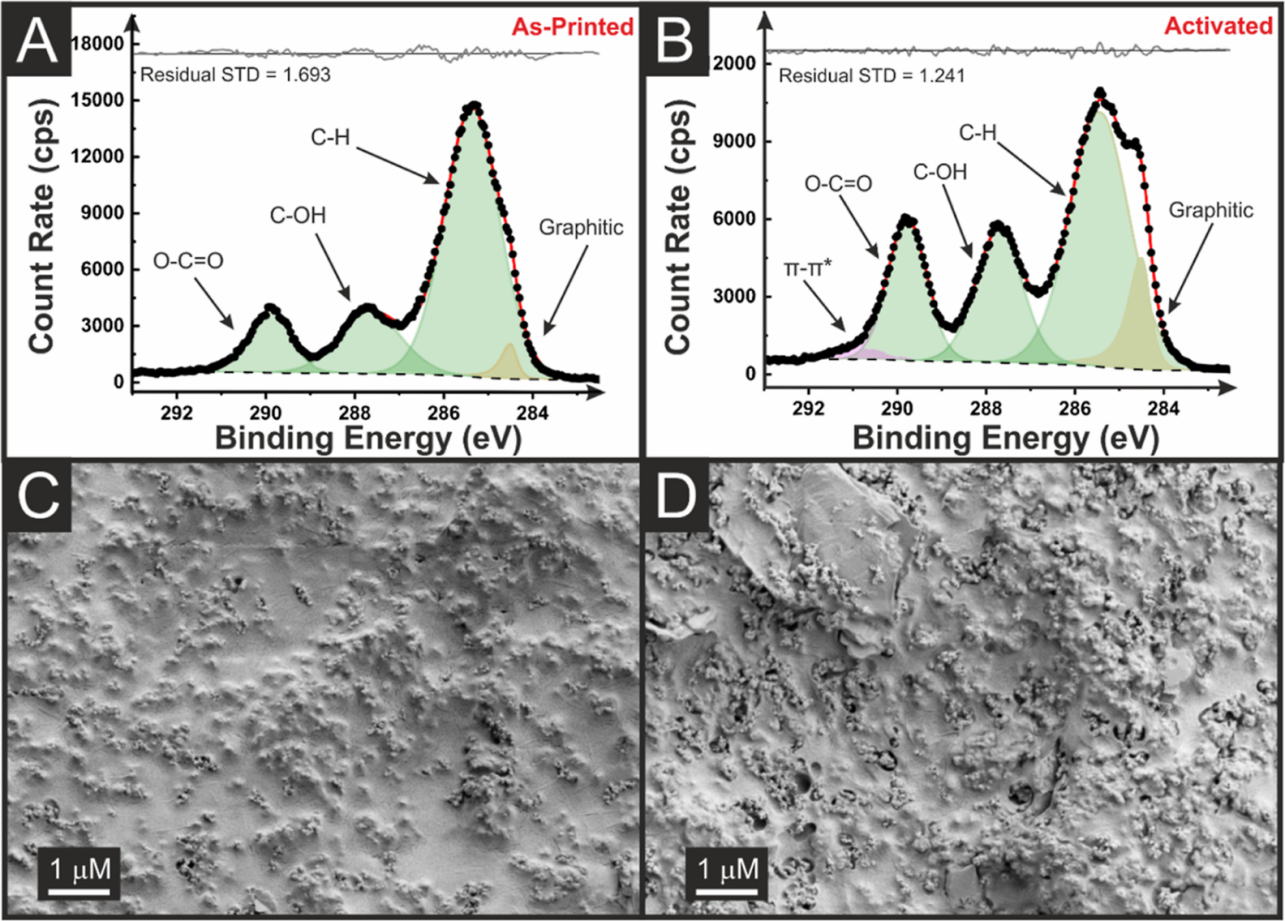
XPS C 1s data for the as-printed (A) and the (B) activated graphite/CB electrode, which shows an increase in the graphitic carbon peak following activation. SEM surface images of the as-printed (C) and the (D) activated graphite/CB electrode.

The chemical composition of the printed AMEs was examined using X-ray photoelectron spectroscopy (XPS) and scanning electron microscopy (SEM), before and after electrochemical activation. The as-printed and activated AME C 1s spectra are shown in Figure 2A,B, respectively. The nonactivated AME C 1s spectrum, Figure 2A, shows three carbon environments corresponding to the C–C/C–H, C–O, and C=O bonding found within PLA and castor oil. It is noted that the C–C/C–H peak found at ∼285 eV has a much larger intensity than the other peaks, which is consistent with the chemical structure of castor oil and is in agreement with XPS data reported previously for filaments combining castor oil and PLA.^21^ Additionally, in Figure 2A, to obtain adequate fitting, an asymmetric peak was required at 284.5 eV which is assigned to the X-ray photoelectron emission by graphitic carbon.^30,31^ This peak was not required in previously reported work on only CB/castor oil filaments and suggests that the graphite flakes could penetrate the print surface, where the CB was mostly embedded below the range probed by XPS (i.e., a few nm). In the activated AME C 1s spectrum, Figure 2B, an increase in the intensity of the graphitic carbon peak at 284.5 eV is observed, indicating that the activation process has effectively stripped the surface nonconductive material away, making the CB available to the range of XPS. Additionally, for adequate fitting of the activated C 1s spectrum, a symmetric peak was required at ∼291 eV, assigned to the π–π* transitions within the large amount of graphitic carbon that is now exposed.^30,31^

Overall, these data provide evidence for the electrochemical activation procedure successfully removing surface PLA to reveal increased amounts of conductive graphitic material below, which should improve the electrochemical performance of the AME toward inner-sphere molecules. SEM images from before and after the electrochemical activation, Figure 2C,D, respectively, further support this conclusion. In Figure 2C, a smooth texture corresponds to the non-conductive PLA with the clear presence of graphite flakes and some CB penetrating the surface. For the activated sample, Figure 2D, there are significant voids in the surface of the PLA, where it has been stripped from the surface, allowing electrochemically active species access to larger amounts of conductive carbon. The Supporting Information brings the SEM images of the CB-only filament, where the same aspects are observed before and after activation (Figure S1A,B), but with the absence of the flakes attributed to the graphite inserted in the new filament.

### Electrochemical Characterization of AMEs

The electrochemical performance of the AMEs printed with the graphite/CB filament was tested using electrodes 3D-printed as a simple lollipop-shaped design, Figure 1D that ensures the electrode connection length is short and keeps the electrochemical response consistent.^32^ A summary of the findings from all of the electrochemical characterization can be found in Table 1, highlighting the peak cathodic current and the peak-to-peak separation (Δ*E*_p_) obtained for [Ru(NH_3_)_6_]^3+^ (1 mM in 0.1 M KCl) at 25 mV s^–1^ scan rate, the calculated *k*^0^ and *A*_e_, along with the solution resistance (*R*_s_) and charge-transfer resistance (*R*_ct_) from electrochemical impedance spectroscopy in a solution of ferri/ferrocyanide ([Fe(CN)_6_]^3–/4–^) (1 mM in 0.1 M KCl) for the graphite AME and two benchmark filaments.

**Table 1:** Comparisons of the Various Electrochemical Parameters, Namely, Cathodic Peak Currents (−*I*_p_^c^), Peak-To-Peak Separations (Δ*E*_p_), Heterogeneous Electron Transfer (*k*^0^), Electrochemically Active Area (*A*_e_), EIS Charge Transfer Resistance (*R*_ct_) and Solution Resistance (*R*_s_) for the Commercial (Protopasta) and the Bespoke Carbon Black and Graphite Filaments^a^

parameter	commercial	CB only	graphite/CB
-*I*_p_^c^ (μA)^b^	65.8 ± 3.5	86.7 ± 6.9	89.1 ± 1.8
Δ*E*_p_ (mV)^b^	238 ± 5	116 ± 8	131 ± 11
*k*^0^ (cm s^–1^)^c^	(0.30 ± 0.03) × 10^–3^	(1.57 ± 0.18) × 10^–3^	(1.26 ± 0.23) × 10^–3^
*A*_e_ (cm^2^)^c^	0.47 ± 0.02	0.65 ± 0.04	0.66 ± 0.01
*R*_ct_ (Ω)^d^	2842 ± 458	475 ± 89	609 ± 166
*R*_s_ (Ω)^d^	768 ± 96	177 ± 23	155 ± 15

a The uncertainties are the standard deviations across three different AME measurements.

b Extracted from cyclic voltammetry at 25 mV s^–1^ in [Ru(NH_3_)_6_]^3+^ (1 mM in 0.1 M KCl).

c Calculated using [Ru(NH_3_)_6_]^3+^ cyclic voltammetric scan rate study performed between 5 and 500 mV s^–1^.

d Extracted from Nyquist plots of EIS experiments in [Fe(CN)_6_]^3–/4–^ (1 mM in 0.1 M KCl). All measurements were performed with a nichrome wire CE and Ag|AgCl (3 M KCl) RE.

**Figure 3:**
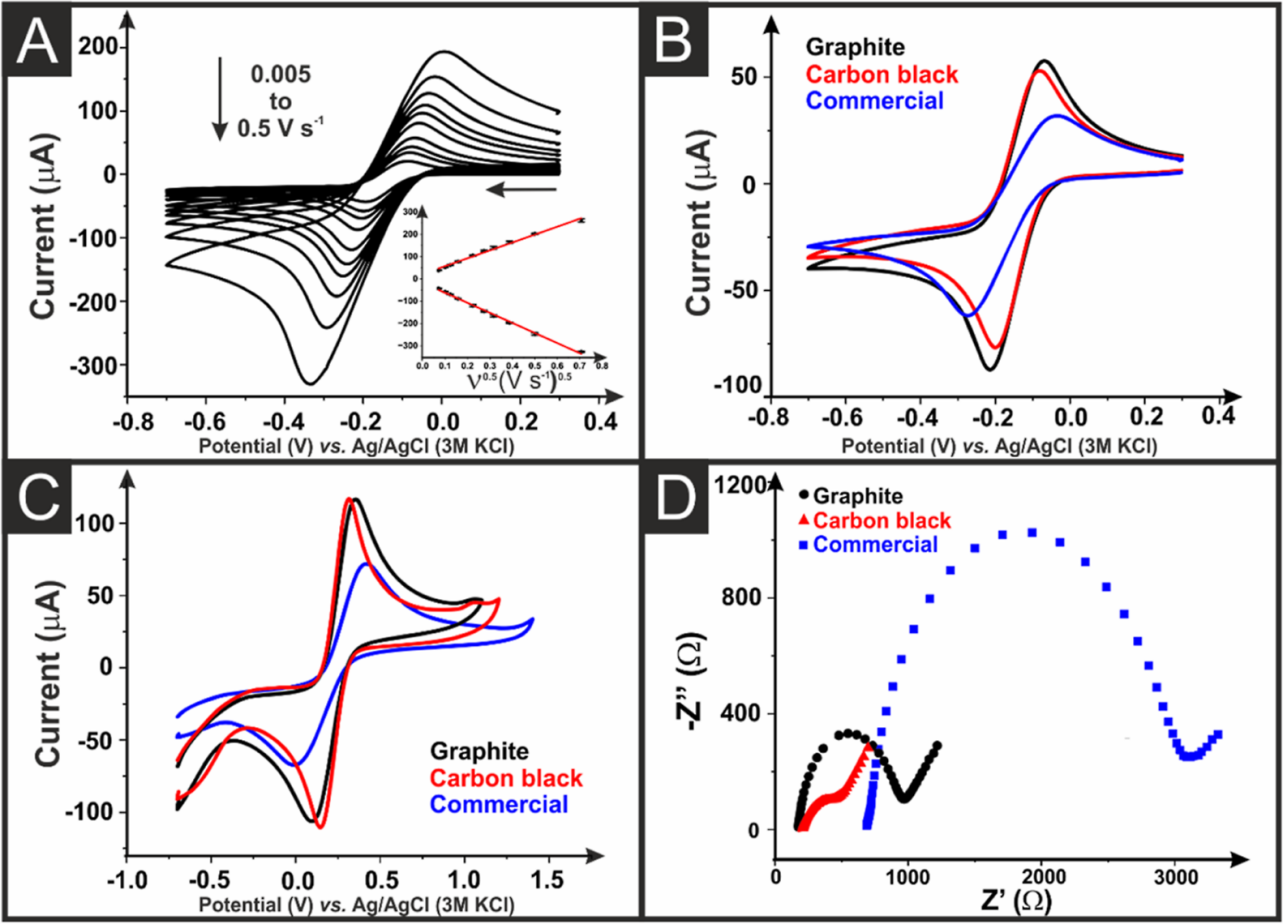
(A) Scan rate study (5–500 mV s^–1^) with 1 mM/0.1 M KCI [Ru(NH_3_)_6_]^3+^ performed using graphite/CB as WE, nichrome coil CE, and Ag|AgCl as RE. The Randles–Ševčík plot is also presented inset. (B) Cyclic voltammograms (25 mV s^–1^) of [Ru(NH_3_)_6_]^3+^ comparing graphite/CB (black) with CB only (red) and the commercial (blue) AMEs. (C) Cyclic voltammograms (25 mV s^–1^) of 1 mM/0.1 M KCI [Fe(CN)_6_]^3–/4–^ comparing the activated graphite/CB with CB only and the commercial AMEs. (D) EIS Nyquist plots of [Fe(CN)_6_]^3–/4–^ were compared with the activated graphite/CB with CB only and the commercial AMEs.

Initially, the as-printed graphite/CB AMEs were tested using cyclic voltammetric scan rate studies against the near-ideal outer-sphere redox probe [Ru(NH_3_)_6_]^3+^ (1 mM in 0.1 M KCl), Figure 3A. This allows for the best determination of the heterogeneous electrochemical rate constant, *k*^0^, and the real electrochemical surface area of the AME, *A*_e_.^33,34^ A comparison of the CV response to [Ru(NH_3_)_6_]^3+^ at 25 mV s^–1^ from the bespoke graphite/CB filament, the commercially purchased CB/PLA filament, and a bespoke, previously published,^21^ CB/rPLA filament is presented in Figure 3B. It is important to compare to a bespoke filament of the same conductive filler wt % comprising only CB to elucidate the effect the graphite has on the system. It can be seen in Figure 3B and Table 1 that the mixed graphite/CB filament performs well compared to the CB/rPLA filament and is significantly better than the commercial alternative CB/PLA in terms of the *k*^0^ and *A*_e_ values obtained.

Activated AMEs made from graphite/CB/rPLA, CB/rPLA, and commercially purchased CB/PLA were tested against 1 mM [Fe(CN)_6_]^3–/4–^ (0.1 M KCl). The response measured at 25 mV s^–1^ represented in Figure 3C confirms that the graphite/CB/rPLA filament performed well compared to the CB/rPLA filament and was significantly better than the commercial CB/PLA filament. Figure 3D shows the Nyquist plot obtained from EIS measurements (100 000–0.1 Hz) in 1 mM [Fe(CN)_6_]^3–/4–^ (0.1 M KCl). Once again, the graphite/CB/rPLA filament performed well with an *R*_ct_ of 609 ± 166 compared to 2842 ± 458 for the commercial filament. It can be seen from Figure 3D and Table 1 that both in-house-made filaments show excellent *R*_s_ values of 155 ± 15 and 177 ± 23 for the graphite/CB/rPLA and CB/rPLA AMEs, respectively. These values were significantly reduced from the commercial filament, which produced an *R*_s_ value of 768 ± 96 Ω.

The characterization of the graphite/CB/rPLA filament indicates the electrochemical performance is vastly improved when compared to the commercially available filament and close to the performance of the bespoke lab-made CB/rPLA filament while replacing 40 wt % of the conductive filler with a significantly lower cost material. Based on the prices from the Super P CB supplier, graphite powder is approximately 12 times cheaper than CB, offering a significant improvement on the AME cost when used. Overall, the cost of producing a single electrode using the graphite/CB composite was £0.05, reduced from £0.09 for an AME produced using only the bespoke CB filament.

### Electroanalytical Determination of Oxalate

Figure S2A-C shows the design of the additive manufactured electrochemical cell used in this work, where the three electrodes are inserted into the removable lid, allowing them to be washed and reused after each analysis, saving cost to the user. The AME working electrode has been designed to be removable from the system, allowing for a new AME to be used on each analysis, avoiding sample contamination and any issues with solution ingress.^17^ With the design of an appropriate cell, the next challenge is applying the AMEs toward detecting oxalate. According to the literature, the electrochemical oxidation of oxalate involves a two-electron mechanism to produce CO_2_,^35^ as shown in Figure S3.

**Figure 4:**
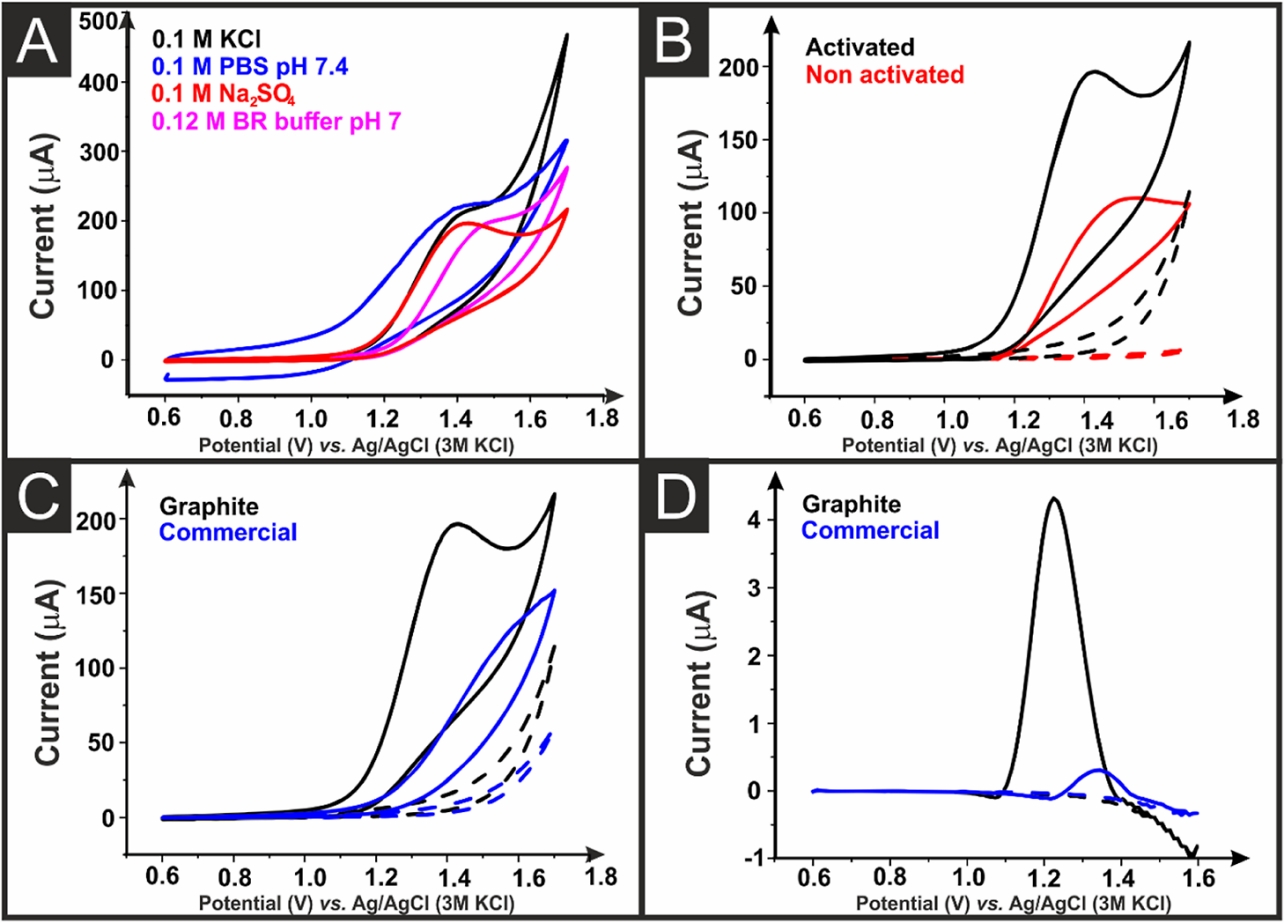
(A) Cyclic voltammograms (50 mV s^–1^) of 1 mM oxalate in different supporting electrolytes recorded at the graphite/CB as WE, the nichrome coil CE, and Ag|AgCl as RE. (B) Cyclic voltammograms (50 mV s^–1^) of 1 mM oxalate (solid lines) in 0.1 M Na_2_SO_4_ electrolyte solution (dashed lines) recorded at graphite/CB AMEs with (black lines) and without (red lines) electrochemical activation. (C) Cyclic voltammograms (50 mV s^–1^) of 1 mM oxalate in 0.1 M Na_2_SO_4_ electrolyte solution were recorded at activated graphite/CB (black) and commercial (blue) AMEs. (D) DPV of 100 μM oxalate in 0.1 M Na_2_SO_4_ electrolyte solution was recorded at activated graphite/CB and commercial AMEs. Step potential: 10 mV. Amplitude: 50 mV.

Figure 4A shows the comparison in the cyclic voltammetric responses obtained for oxalate (1 mM) in KCl, PBS (pH 7.4), Britton Robinson (BR) buffer (pH 7), and Na_2_SO_4_ using a graphite/CB electrode. A very well-defined peak was observed in Na_2_SO_4_ (0.1 M). Figure 4B shows an increase in the oxidation peak current of oxalate when AME is activated. The peak oxidation current increased from 41.9 ± 1.9 to 88.9 ± 3.5 μA upon electrochemical activation. Figure 4C,D shows the cyclic voltammetric and differential pulse voltammetry (DPV) responses to oxalate, 1 mM and 100 μM, respectively, at an electrochemically activated AME printed from the graphite/CB filament and the comparison to the commercial CB conductive filament. This highlights how the oxidation peaks are greatly increased in magnitude and found at lower peak potentials when using the bespoke graphite/CB filament. The commercial conductive filament produced a DPV peak current of 0.4 ± 0.1 μA, which increased to 4.1 ± 0.8 μA when the graphite/CB filament.

**Figure 5:**
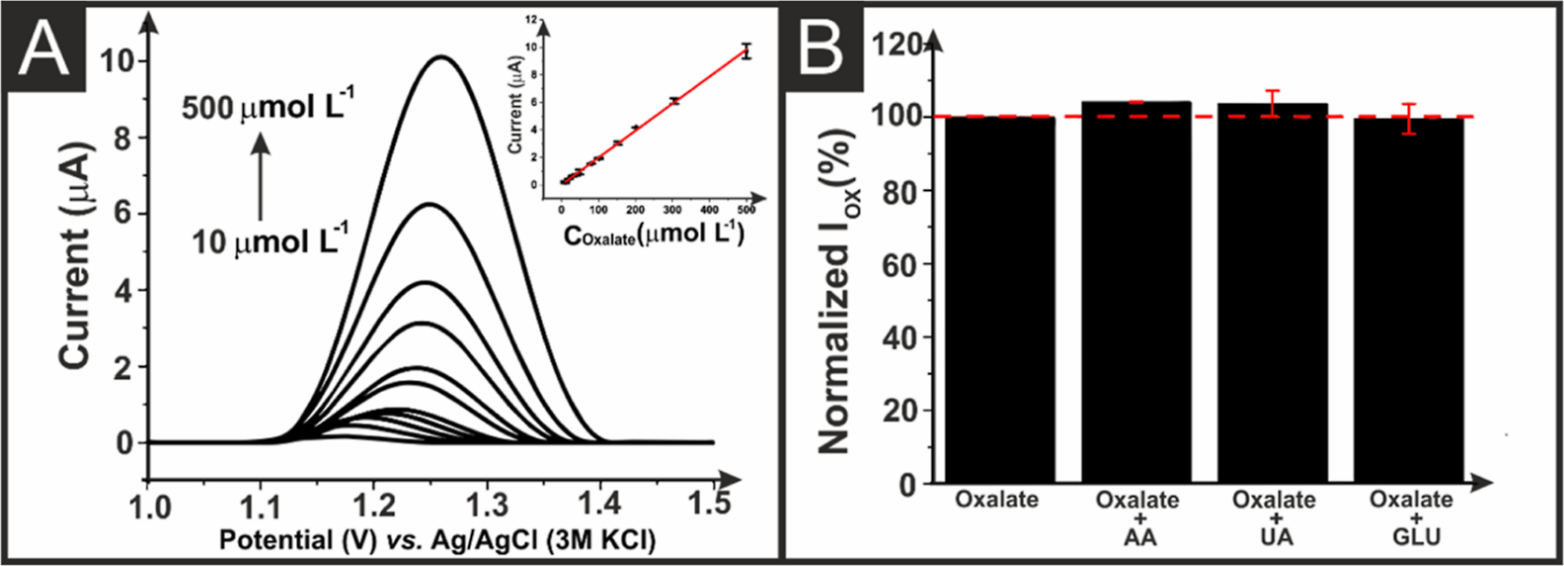
(A) DPV of oxalate in different concentrations (10 to 500 μM) in 0.1 M Na_2_SO_4_ recorded at activated graphite/CB AME and the respective calibration curve inset. Step potential: 3 mV. Amplitude: 60 mV. (B) Normalized peak current (%) of 100 μM oxalate in the presence of 200 μM uric acid, ascorbic acid, and glucose in 0.1 M Na_2_SO_4_.

First, DPV parameters were optimized, as shown in Figures S4 and S5, with the best results obtained using a step potential of 3 mV and an amplitude of 60 mV. Then, optimized DPV was used to produce the analytical curves for the detection of oxalate using AMEs printed from both commercial conductive filament, Figure S6, and the bespoke graphite/CB filament, Figure 5A. A summary of the results obtained can be seen in Table 2. It can be seen that the peak oxidation current increased linearly with oxalate concentration in both cases with the graphite/CB AME producing a wide linear range of 10–500 μM (*I* (μA) = 0.0196 *C*_Oxalate_ (μM) + 0.0223; *R* = 0.9996), compared to 80–500 μM (*I* (μA) = 0.0045 *C*_Oxalate_ (μM) – 0.1628; *R* = 0.9947) for the commercial AME. The graphite/CB filament gave improved electroanalytical results in all cases, with a sensitivity of 0.0196 μA/μM, limit of detection (LOD) of 5.7 μM, and limit of quantification (LOQ) of 18.8 μM. These results compared well with other electrochemical sensors reported within the literature for oxalate detection, as summarized in Table S2.

**Table 2:** Comparison of the Analytical Parameters Obtained for Oxalate Determination Utilizing the Commercial CB/PLA and the Bespoke Graphite/CB AMEs

parameter	commercial	graphite/CB
linear range (μM)	80–500	10–500
sensitivity (μA/μM)	0.0045	0.0196
LOD (μM)	41.2	5.7
LOQ (μM)	135.9	18.8

The repeatability of oxalate measurements with graphite/CB AME was then tested, Figure S7, with a relative standard deviation of 7.1% obtained over the course of 8 measurements. Furthermore, the selectivity of the electroanalytical platform was assessed by analyzing oxalate in the presence of twice the concentration of common interferents, such as ascorbic acid, uric acid, and glucose, Figure 5B. It can be seen that acceptable results were obtained in the presence of the interferents, with a minor increase in peak oxidation currents of ascorbic acid and uric acid.

The detection of oxalate was then performed into a spiked synthetic urine sample, Figure S8, to mimic the electroanalytical sensing platform use within a real-world application. Using the cell designed in Figure S2, a sample could be added to the container, filling the line. The electrodes were placed within the marked slots and the lid was screwed into place to ensure a reproducible experimental setup. A good recovery value of 104% was obtained, highlighting the applicability of this AM electroanalytical sensing platform for detecting oxalate in urine.

## Conclusions

This work has presented the production and characterization of a graphite/CB composite filament utilizing recycled PLA and castor oil as a plasticizer. The filament was physiochemically characterized through TGA, XPS, and SEM to provide evidence for successfully incorporating these materials into an additive manufacturing filament. The graphite/CB filament was electrochemically characterized against a previously reported filament containing only CB and a commonly used commercial conductive filament. The cost of printing AMEs from the graphite/CB filament was reduced to £0.05 per electrode, compared to £0.09 for the previously reported CB-only filament. The graphite/CB performed well compared to the CB-only filament and outperformed the commercial filament, producing a *k*^0^ of (1.26 ± 0.23) × 10^–3^ cm s^–1^ and AME resistance of only 155 ± 15 Ω, compared to (0.30 ± 0.03) × 10^–3^ cm s^–1^ and 768 ± 96 Ω for the commercial AME, respectively.

A bespoke AM cell was designed to allow for the simple and quick determination of oxalate within urine samples. This was used to test the electroanalytical performance of the graphite/CB AMEs, where a sensitivity of 0.0196 μA/μM, LOD of 5.7 μM, and LOQ of 18.8 μM was obtained. The graphite/CB AMEs showed excellent reproducibility and selectivity against excess concentrations of common interferents. Additionally, the cell was tested for the detection of oxalate within a spiked synthetic urine sample, obtaining recoveries of 104%. This work highlights how through the use of mixed material composites, excellent electrochemical performance can be obtained at a reduced material cost while also improving the sustainability of the system.

## Supporting Information Available

TGA onset temperatures and filler % for all components of recycled filament; comparison of the bespoke AME with other electrochemical sensors; SEM images of the CB only AME; images of the AM oxalate detection cell; oxalate oxidation mechanism; DPV optimization of step potential and amplitude; analytical curve for oxalate in the CB only AME; oxalate repeatability measurements; standard addition curve for oxalate in synthetic urine sample; physiochemical and electrochemical characterization (PDF)
