# Supplementary material for: Mixed Graphite/Carbon Black Recycled PLA Conductive Additive Manufacturing Filament for the Electrochemical Detection of Oxalate
Source: Anal Chem. 2023 Sep 28;95(40):15086–93. doi: 10.1021/acs.analchem.3c03193 (PMC10568530; doi:10.1021/acs.analchem.3c03193)
Supplement: Supplementary file 1 — ac3c03193_si_001.pdf [file ac3c03193_si_001.pdf]

Supporting Information for:

Mixed graphite/carbon black recycled PLA conductive additive manufacturing filament for the electrochemical detection of oxalate

Iana V.S. Arantes,[1,2] Robert D. Crapnell,[1] Elena Bernalte,[1] Matthew J. Whittingham,[1] Thiago R.L.C. Paixão,[2] and Craig E. Banks[1*]

[1]*Faculty of Science and Engineering, Manchester Metropolitan University, Chester Street, M1 5GD, United Kingdom.*

[2]*Departmento de Química Fundamental, Instituto de Química, Universidade de São Paulo, São Paulo, SP, 05508-000, Brazil.*

[*]To whom correspondence should be addressed.

E-mail: c.banks@mmu.ac.uk; Tel: +44(0)1612471196

Table of Content

Table S1. TGA onset temperatures and filler % for all components of recycled filament. The uncertainties in Onset Temperature and Filler Content are the standard deviations of three repeat measurements……………………………………………………………...….S4

Table S2. Analytical parameters obtained from bespoke filament AMEs compared to other electrodes found in the literature for oxalate determination……………………………..…S4

Figure S1. SEM surface images for the **(A)** as-printed and the **(B)** activated CB/only AME…S4

Figure S2. CAD designs for the production of the oxalate detection cell **(A)** Fully assembled cell, **(B)** Cut-through cell showing it filled with solution, and **(C)** Dissembled view showing the electrodes in the lid………………………………………………………………………S5

Figure S3. Schematic representation of the electrochemical oxidation of oxalate…………S5

Figure S4. (A) Differential pulse voltammetry of 100 μM oxalate in 0.1 M Na_2SO_4 electrolyte solution were recorded at the graphite/CB AME using different step potential values and **(B)** the respective peak currents. Amplitude: 50 mV……………………………………………..S6

Figure S5. (A) Differential pulse voltammetry of 100 μM oxalate in 0.1 M Na_2SO_4 electrolyte solution were recorded at the graphite/CB AME using different amplitude values and **(B)** the respective peak currents. Step potential: 10 mV……………………………………………..…S6

Figure S6. (A) Differential pulse voltammetry of oxalate in different concentrations (80 to 500 μM) in 0.1 M Na_2SO_4 recorded at activated commercial CB/PLA AME and **(B)** the respective calibration curve. Step potential: 3 mV. Amplitude: 60 mV…………………………………S7

Figure S7. Repeatability test (n=8) performed under the optimized differential pulse voltammetry conditions to detect 100 μM oxalate in 0.1 M Na_2SO_4 at the graphite/CB AME. Amplitude: 60 mV. Step potential: 3 mV……………………………………………………..S7

Figure S8. (A). Differential pulse voltammetry measurements of a spiked synthetic urine sample (500 μM) (red line) diluted ~20-fold in 0.1 M Na_2SO_4 with subsequent additions of oxalate standard solutions (10 to 50 μM) (black lines), and **(B)** the respective calibration curve. Step potential: 3 mV. Amplitude: 60 mV……………………………………………………..S8

Details of the physiochemical characterization and electrochemical experiments……………S9

REFERENCES……………………………………………………………………….S11

Table S1. TGA onset temperatures and filler % for all components of recycled filament. The uncertainties in Onset Temperature and Filler Content are the standard deviations of three repeat measurements.

Material	Onset Temperature (°C)	Conductive Filler (wt %)
**rPLA**	304 ± 4	-
**Castor Oil**	250 ± 3	-
**Graphite/CB Filament**	283 ± 4	24 ± 1

Table S2. Analytical parameters obtained from bespoke filament AMEs compared to other electrodes found in the literature for oxalate determination.

Electrode	Technique	Linear range (µM)	LOD (µM)	Reference
**BPPG electrode**	CV	500 − 3500	0.70	1
**SiO_2/C/CoPc pressed disk electrodes**	Chronoamperometry	39.8 − 460	0.58	2
**AuNP/PPy/ rGO modified GCE**	Chronoamperometry	50 − 7000	20	3
**g-C_3N_4/CPE**	SWV	1 − 1000	0.75	4
**Graphite/Ag/AgCl nanocomposite CPE**	DPV	10 − 750	3.7	5
**CuS nanosphere modified GCE**	Chronoamperometry	10 − 700	35.6	6
**Graphite/CB/rPLA AME**	DPV	10 − 500	5.7	This work

BPPG: basal plane pyrolytic graphite. **CoPc**: cobalt phthalocyanine. **AuNP/PPy/rGO**: gold nanoparticle, polypyrrole, and reduced graphene oxide. **GCE**: glassy carbon electrode. **g-C_3N_4**: graphitic carbon nitride. **CPE:** carbon paste electrode. **CB/rPLA**: carbon black and recycled PLA. **AME**: additively manufactured electrode.

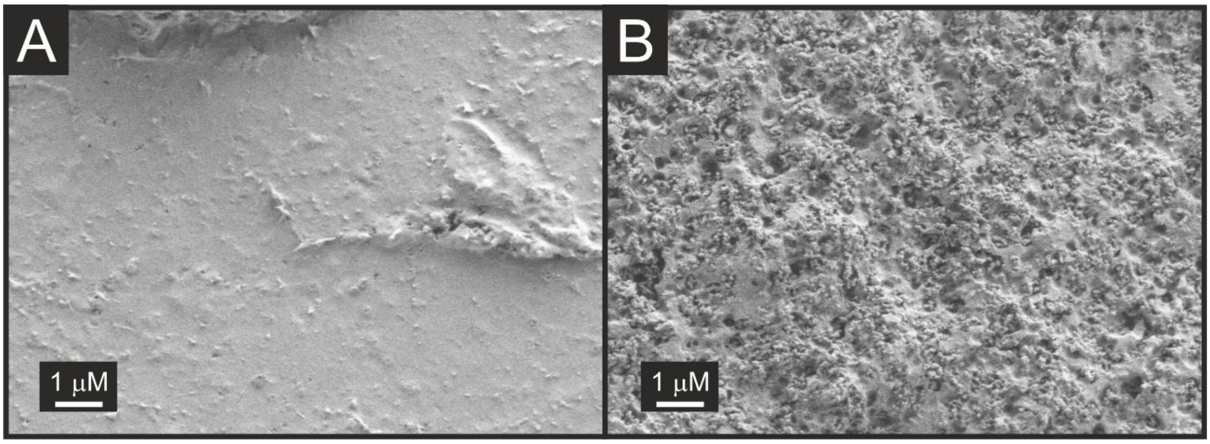

Figure S1. SEM surface images for the **(A)** as-printed and the **(B)** activated CB only AME.

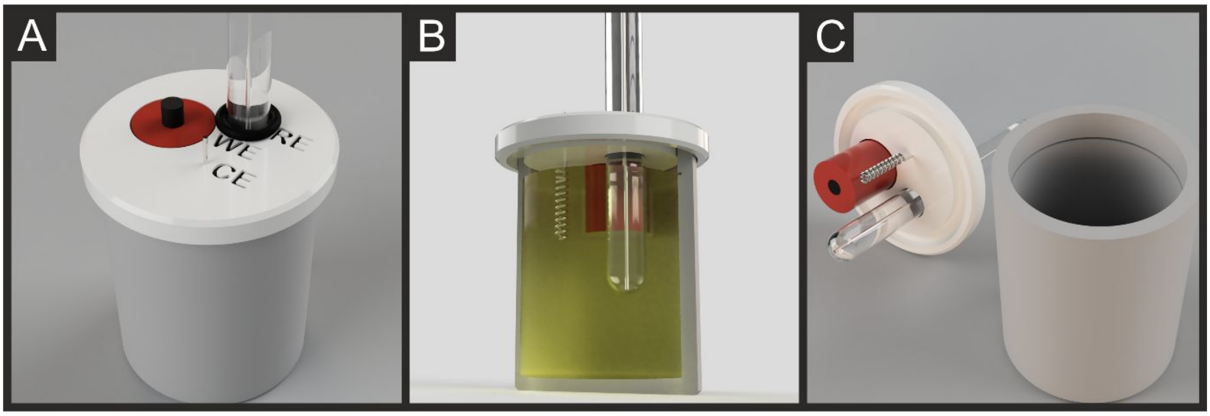

Figure S2. CAD designs for the production of the oxalate detection cell **(A)** Fully assembled cell, **(B)** Cut-through cell showing it filled with solution, and **(C)** Dissembled view showing the electrodes in the lid.

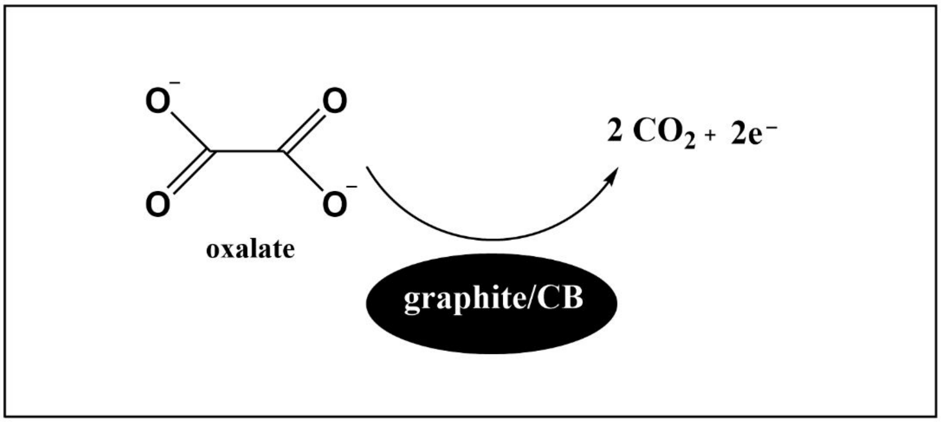

Figure S3. Schematic representation of the electrochemical oxidation of oxalate.

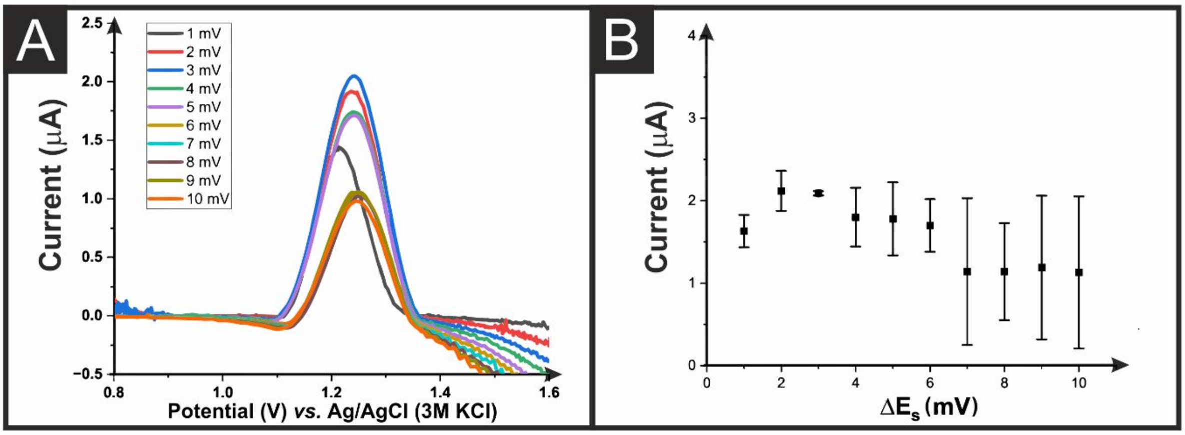

Figure S4. (A) Differential pulse voltammetry of 100 μM oxalate in 0.1 M Na_2SO_4 electrolyte solution were recorded at the graphite/CB AME using different step potential values and **(B)** the respective peak currents. Amplitude: 50 mV.

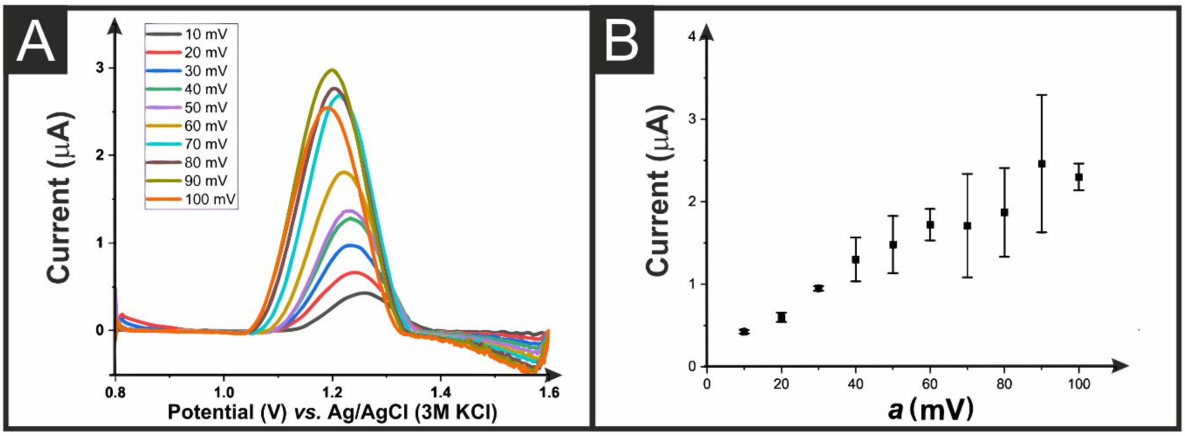

Figure S5. (A) Differential pulse voltammetry of 100 μM oxalate in 0.1 M Na_2SO_4 electrolyte solution were recorded at the graphite/CB AME using different amplitude values and **(B)** the respective peak currents. Step potential: 10 mV.

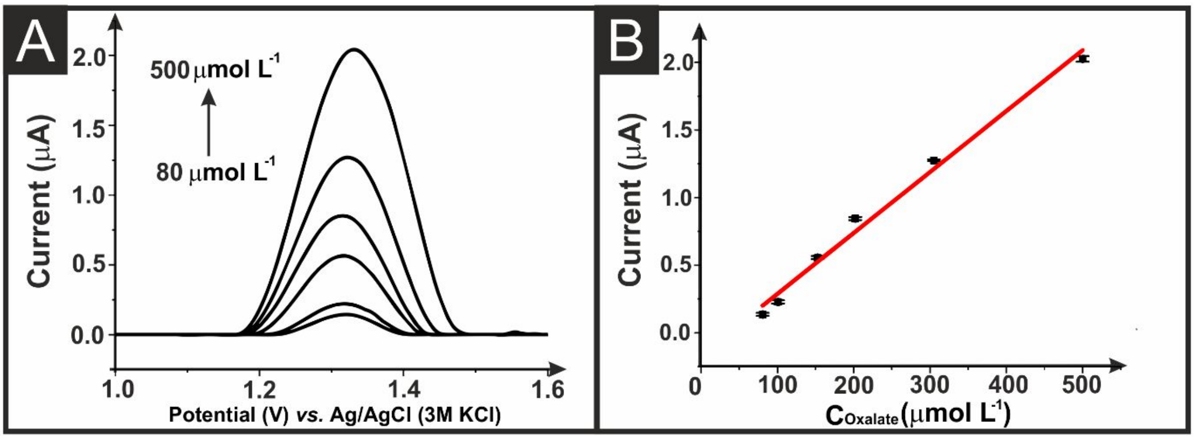

Figure S6. (A) Differential pulse voltammetry of oxalate in different concentrations (80 to 500 μM) in 0.1 M Na_2SO_4 recorded at activated commercial CB/PLA AME and **(B)** the respective calibration curve. Step potential: 3 mV. Amplitude: 60 mV.

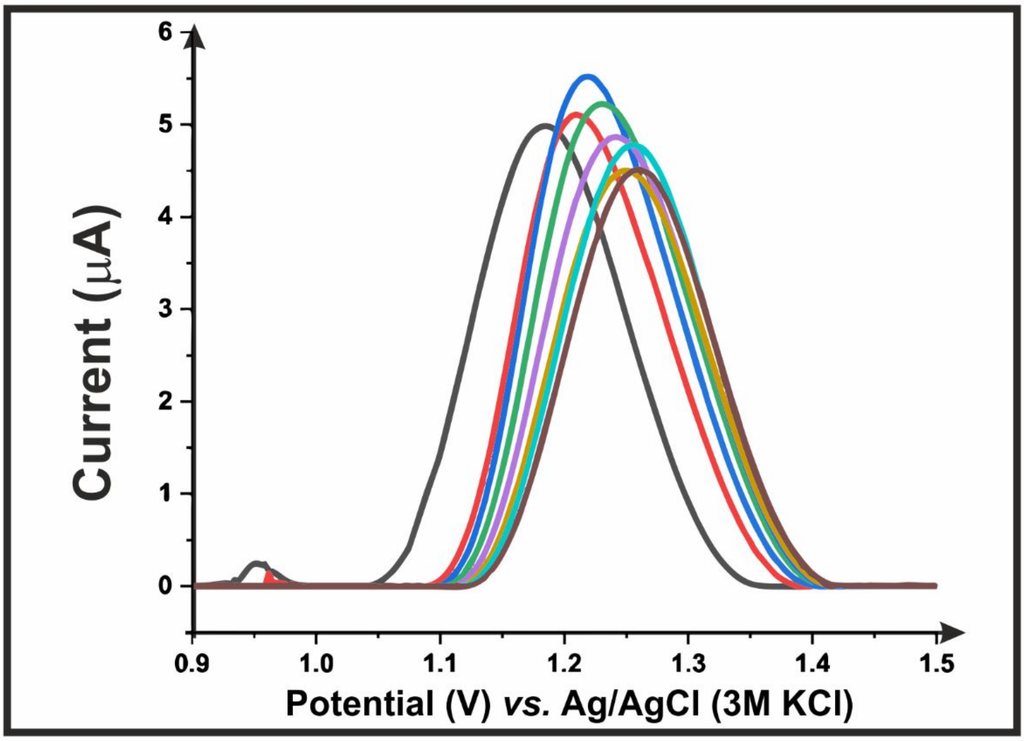

Figure S7. Repeatability test (n=8) performed under the optimized differential pulse voltammetry conditions to detect 100 μM oxalate in 0.1 M Na_2SO_4 at the graphite/CB AME. Amplitude: 60 mV. Step potential: 3 mV.

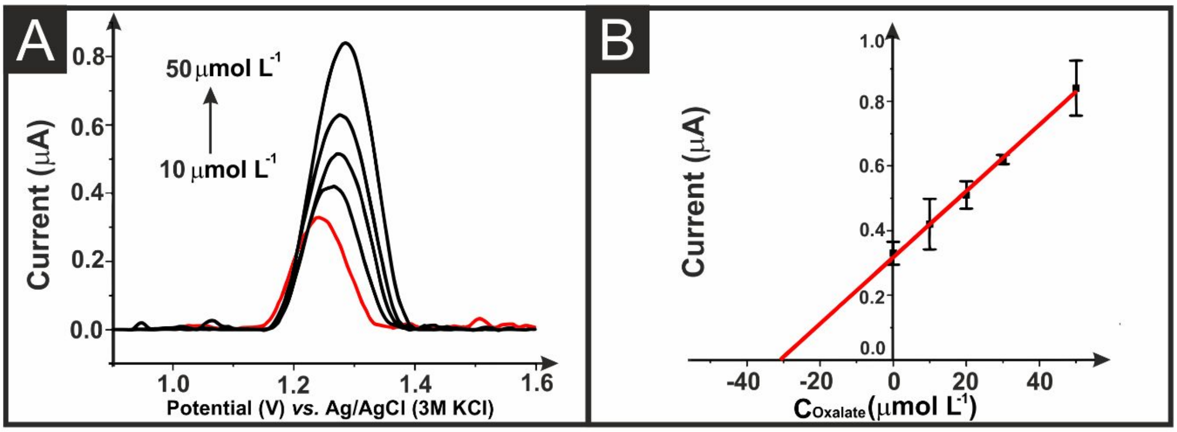

Figure S8. (A). Differential pulse voltammetry measurements of a spiked synthetic urine sample (500 μM) (red line) diluted ~20-fold in 0.1 M Na_2SO_4 with subsequent additions of oxalate standard solutions (10 to 50 μM) (black lines), and **(B)** the respective calibration curve. Step potential: 3 mV. Amplitude: 60 mV.

Physiochemical characterisation

Thermogravimetric analysis (TGA) was performed using a Discovery Series SDT 650 controlled by Trios Software (TA Instruments, DA, USA). Samples were mounted in alumina pans and tested using a ramp profile (10 °C min^{-1}) from 0 – 800 °C under N_2 (100 mL min^{-1}).

X-ray Photoelectron Spectroscopy (XPS) data were acquired using an AXIS Supra (Kratos, UK), equipped with a monochromated Al X-ray source (1486.6 eV) operating at 225 W and a hemispherical sector analyser. It was operated in fixed transmission mode with a pass energy of 160 eV for survey scans and 20 eV for region scans with the collimator operating in slot mode for an analysis area of approximately 700 x 300 µm, the FWHM of the Ag 3d5/2 peak using a pass energy of 20 eV was 0.613 eV. Before analysis, each sample was ultrasonicated for 15 min in propan-2-ol and then dried for 2.5 hours at 60 °C as shown in our unpublished data, to remove excess contamination and minimise the risk of misleading data. The binding energy scale was calibrated by setting the graphitic sp^2 C 1s peak to 284.5 eV; this calibration is acknowledged to be flawed [25], but was nonetheless used in the absence of reasonable alternatives, and because only limited information was to be inferred from absolute peak positions.

Scanning Electron Microscopy (SEM) measurements were recorded on a Supra 40VP Field Emission (Carl Zeiss Ltd., Cambridge, UK) with an average chamber and gun vacuum of 1.3×10^{-5} and 1×10^{-9} mbar, respectively. Samples were mounted on the aluminium SEM pin stubs (12 mm diameter, Agar Scientific, Essex, UK). To enhance the contrast of these images, a thin layer of Au/Pd (8 V, 30 s) was sputtered onto the electrodes with the SCP7640 from Polaron (Hertfordshire, UK) before being placed in the chamber.

Raman spectroscopy was performed on a Renishaw PLC in Via Raman Microscope controlled by WiRE 2 software at a laser wavelength of 514 nm.

Electrochemical experiments

All electrochemical measurements were performed on an Autolab 100N potentiostat controlled by NOVA 2.1.6 (Utrecht, the Netherlands). The electrochemical characterisation of the bespoke filament and comparison to the benchmarks were performed using a lollipop design (Ø 4 mm disc with 8 mm connection length and 2 x 1 mm thickness) electrodes alongside an external commercial Ag|AgCl (3M KCl) reference electrode and a nichrome wire counter electrode. All solutions of hexaamineruthenium (III) chloride were prepared using

deionised water of resistivity not less than 18.2 MΩ cm from a Milli-Q system (Merck, Gillingham, UK) and thoroughly degassed for 15 min prior to any electrochemical experiments.

Electrochemical activation of the AMEs in NaOH was performed before all electrochemical experiments described in the literature [12]. Briefly, the AMEs were connected as the working electrode in conjunction with a nichrome wire coil counter and Ag|AgCl (3 M KCl) reference electrode and placed in a solution of NaOH (0.5 M). Chronoamperometry was used to activate the AMEs by applying a set voltage of + 1.4 V for 200 s, followed by applying - 1.0 V for 200 s. The AMEs were then thoroughly rinsed with deionised water and dried under compressed air before further use.

REFERENCES

(1) Šljukić, B.; Baron, R.; Compton, R. G. Electrochemical Determination of Oxalate at Pyrolytic Graphite Electrodes. *Electroanalysis* **2007**, *19* (9), 918–922. https://doi.org/10.1002/elan.200703852.

(2) Rahim, A.; Barros, S. B. A.; Arenas, L. T.; Gushikem, Y. In Situ Immobilization of Cobalt Phthalocyanine on the Mesoporous Carbon Ceramic SiO2/C Prepared by the Sol-Gel Process. Evaluation as an Electrochemical Sensor for Oxalic Acid. *Electrochim. Acta* **2011**, *56* (3), 1256–1261. https://doi.org/10.1016/j.electacta.2010.11.009.

(3) Sharma, P.; Radhakrishnan, S.; Jayaseelan, S. S.; Kim, B. S. Non-Enzymatic Electrochemical Oxidation Based on AuNP/PPy/RGO Nanohybrid Modified Glassy Carbon Electrode as a Sensing Platform for Oxalic Acid. *Electroanalysis* **2016**, *28* (10), 2626–2632. https://doi.org/10.1002/elan.201600266.

(4) Alizadeh, T.; Nayeri, S.; Hamidi, N. Graphitic Carbon Nitride (g-C3N4)/Graphite Nanocomposite as an Extraordinarily Sensitive Sensor for Sub-Micromolar Detection of Oxalic Acid in Biological Samples. *RSC Adv.* **2019**, *9* (23), 13096–13103. https://doi.org/10.1039/c9ra00982e.

(5) Alizadeh, T.; Nayeri, S. Graphite/Ag/AgCl Nanocomposite as a New and Highly Efficient Electrocatalyst for Selective Electroxidation of Oxalic Acid and Its Assay in Real Samples. *Mater. Sci. Eng. C* **2019**, *100* (May 2018), 826–836. https://doi.org/10.1016/j.msec.2019.03.052.

(6) Venkadesh, A.; Mathiyarasu, J.; Radhakrishnan, S. Electrochemical Enzyme-Free Sensing of Oxalic Acid Using an Amine-Mediated Synthesis of CuS Nanosphere. *Anal. Sci.* **2021**, *37* (7), 949–954. https://doi.org/10.2116/analsci.20P370.
